# Commentary: CD6 As a Potential Target for Treating Multiple Sclerosis

**DOI:** 10.3389/fimmu.2017.01217

**Published:** 2017-09-29

**Authors:** Marta Consuegra-Fernández, Marcos Isamat, Francisco Lozano

**Affiliations:** ^1^Immunoreceptors of the Innate and Adaptive System, Institut d’Investigacions Biomèdiques August Pi i Sunyer, Barcelona, Spain; ^2^Servei d’Immunologia, Centre de Diagnòstic Biomèdic, Hospital Clínic de Barcelona, Barcelona, Spain; ^3^Departament de Biomedicina, Universitat de Barcelona, Barcelona, Spain

**Keywords:** CD6, multiple sclerosis, itolizumab, UMCD6, experimental autoimmune encephalomyelitis, collagen-induced arthritis, cGvHD-induced lupus-like disease

The recently published article by Li et al. reports that DBA/1 mice deficient for the T-cell receptor CD6 (CD6^−/−^) undergo augmented T-cell activation together with reduced activated T-cell survival/proliferation and decreased T_H_1 and T_H_17 polarization, leading to overall attenuated experimental autoimmune encephalomyelitis (EAE) *in vivo* (1). Furthermore, by developing CD6 humanized mice, they also show the effectiveness of a mouse anti-human CD6 (UMCD6) monoclonal antibody (mAb) in treating established EAE without depleting T cells (1). This comes at a time of growing expectation that seems to place CD6 as a global therapeutic target for immune-mediated inflammatory diseases, in line with multiple sclerosis high risk *CD6* gene variants (2). CD6 was selected as a potential target for the humanized anti-human CD6 mAb Itolizumab—currently under clinical investigation in Psoriasis and other autoimmune diseases (3)—based on *in vitro* data at a time when no CD6^−/−^ mice were available.

In their report, Li et al. refer to our work showing increased severity of collagen-induced arthritis (CIA) in CD6^−/−^ mice of C57BL/6 background (4). Accordingly, the authors claim that additional studies will be required to unravel the reasons underlying the apparent differences between different autoimmune models and genetically different mouse strains in the etiopathogenic role of CD6 in autoimmunity development. In this regard, we have observed that CD6^−/−^ C57BL/6 mice undergoing myelin oligodendrocyte glycoprotein peptide (MOG_35–55_)-induced EAE show a non-statistically significant trend to lower clinical score (*p* = 0.1) and weight loss (*p* = 0.052) (Figure 1). Differences in EAE-induction protocols or in the sensitivity of mouse strains to EAE-induction (among others; e.g., differences in microbiota composition and housing conditions) may likely explain the observed quantitative discrepancies between the two studies. Whatever the case, the lack of exacerbated EAE in CD6^−/−^ mice of C57BL/6 background contrasts with the increased severity of CIA (4) and lupus-like disease (5) in the same mouse strain. This would indicate that CD6 could play different roles in different models of autoimmune disease.

**Figure 1:**
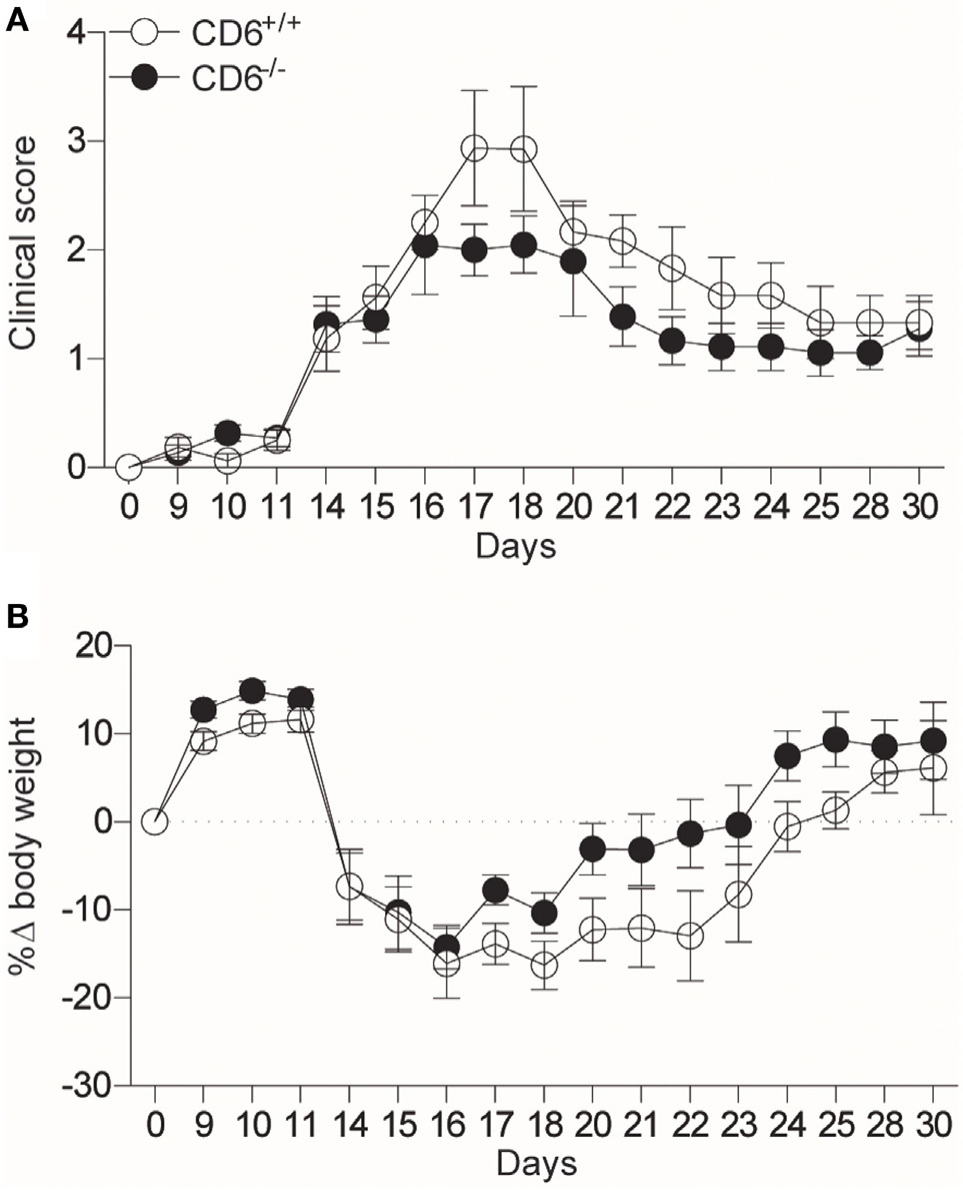
CD6^−/−^ C57BL/6 mice show attenuated experimental autoimmune encephalomyelitis (EAE). **(A)** Clinical scores (*p* = 0.1) and **(B)** body weight loss (*p* = 0.052) of CD6^+/+^ and CD6^−/−^ C57BL/6 mice undergoing MOG_35–55_-induced EAE. Combined results from four experiments (mean ± SEM) are represented (*n* = 40 mice per group). Statistical comparisons were performed by ANOVA, starting from day 14 on (where the clinical signs and weight loss were evidenced).

Li et al. also show attenuated EAE in CD6 humanized DBA/1 mice following treatment with UMCD6, a mAb binding to domain 1 (D1) of CD6 and not blocking CD6–CD166/ALCAM interaction (6), two characteristics shared by Itolizumab. In light of this, the authors hypothesize disruption of CD6 interaction with a still ill-defined D1-binding ligand (6). While this possibility awaits clarification, mAb-induced receptor modulation-related events should also be taken into consideration. Receptor ligation with high affinity and bivalent reagents like anti-CD6 mAbs trigger intracellular signals and/or receptor internalization. Importantly, CD6 is physically associated to the TCR/CD3 complex and other co-stimulatory receptors (CD4, CD5, CD8, and CD28) and/or intracellular effectors (Lck, Fyn, SLP-76, and Syntenin) (7). Consequently, mAb-induced receptor crosslinking and/or down-modulation of associated signaling effectors would also impact *in vivo* effects. Indeed, reductions in absolute CD3^+^, CD4^+^, and CD8^+^ counts have been reported during immunological evaluation of patients just after treatment with non-cytotoxic Itolizumab (8). According to Li et al., UMCD6 does not alter peripheral CD4^+^ and CD8^+^ lymphocyte percentages at day 7 post-treatment. This time scale enables surface re-expression of high turnover receptors (i.e., CD3) (9). Therefore, serial phenotyping shortly after UMCD6 infusion would possibly reveal restoration of self-tolerance by transient co-modulation of relevant lymphocyte receptors/effectors (9).

As it stands, the ongoing discussion highlights the need to broaden our knowledge on CD6 biology, as illustrated by the unexpected opposing observations from CD6^−/−^ mice undergoing different autoimmune disorders. Similarly, detailed mechanistic studies are needed when assaying mAb-based therapies to univocally assign them solely to CD6, irrespective of the encouraging results already reported in preliminary clinical studies (3).

## Author Contributions

MC-F wrote the manuscript and generated experimental data. MI and FL wrote the manuscript.

## Conflict of Interest Statement

The authors declare that the research was conducted in the absence of any commercial or financial relationships that could be construed as a potential conflict of interest.
